# A combination of gemcitabine and 5-fluorouracil in advanced pancreatic cancer, a report from the Italian Group for the Study of Digestive Tract Cancer (GISCAD)

**DOI:** 10.1038/sj.bjc.6690568

**Published:** 1999-07

**Authors:** S Cascinu, R R Silva, S Barni, R Labianca, L Frontini, E Piazza, G Pancera, P Giordani, L Giuliodori, M A Pessi, V Fusco, G Luporini, R Cellerino, G Catalano

**Affiliations:** Division of Medical Oncology, Azienda Ospedaliera ‘Ospedale S Salvatore’, 61100 Pesaro, Italy; Medical Oncology Unit, Ospedale di Fabriano, Fabriano, 60044 Fabriano, Italy; Department of Radiation Oncology, Ospedale S Gerardo, 20052 Monza, Italy; Division of Medical Oncology, Ospedale S Carlo Borromeo, 20153 Milano, Italy; Medical Oncology Unit, Ospedale S Paolo, 20153 Milano, Italy; Division of Medical Oncology, Ospedale L Sacco, 20157 Milano, Italy; Medical Oncology, Casa di Cura Igea, 20153 Milano, Italy; Division of Medical Oncology, Ospedale Civile, 15100 Alessandria, Italy; Division of Medical Oncology, Ancona University, 60020 Ancona, Italy

**Keywords:** pancreatic cancer, intensive chemotherapy, palliation

## Abstract

In a randomized clinical trial, gemcitabine (GEM) was more effective than 5-fluorouracil (5-FU) in advanced pancreatic cancer patients. GEM and 5-FU have different mechanisms of action and their combination, from a theoretical point of view, could result in a higher activity. To test activity and feasibility of such a combination, a multi-institutional phase II study was initiated in November 1996 by the Italian Group for the study of Digestive Tract Cancer (GISCAD). Primary objectives of this study were to determine the activity in terms of response rate and clinical benefit, while the secondary objective was toxicity. According to the optimal two-stage phase II design, 54 patients were enrolled. Schedule was: GEM 1000 mg m^−2^ intravenous (i.v.), and 5-FU 600 mg m^−2^ bolus i.v. weekly for 3 weeks out of every 4. All the 54 patients were symptomatic (pain, weight loss, dyspepsia). A clinical benefit was obtained in 28 patients (51%) (95% confidence interval (CI) 38–64%). Two patients achieved a partial response and 34 a stable disease. Median survival for all the patients was 7 months. Side-effects were mild: no gastrointestinal or haematological grade 3–4 toxicity (WHO) were recorded. We observed only six episodes of grade 2 (WHO) leukopenia and seven episodes of thrombocytopenia. Although the non-randomized design of this study suggests caution in the interpretation of these data, in consideration of the low incidence of toxicity and the favourable results obtained in terms of clinical benefit, it may be worthwhile to test more active schedules of 5-FU (continuous infusion) in combination with gemcitabine. © 1999 Cancer Research Campaign


*British Journal of Cancer* (1999) **80**(10), 1595–1598
© 1999 Cancer Research Campaign
Article no. bjoc.1999.0568

### A combination of gemcitabine and 5-fluorouracil in advanced pancreatic cancer, a report from the Italian Group for the Study of Digestive Tract Cancer (GISCAD)

**S Cascinu[1], RR Silva[2], S Barni[3], R Labianca[4], L Frontini[5], E Piazza[6], G Pancera[7], P Giordani[1], L Giuliodori[2], MA Pessi[4], V Fusco[8], G Luporini[4], R Cellerino[9] and G Catalano[1]**

[1]Division of Medical Oncology, Azienda Ospedaliera 'Ospedale S Salvatore', 61100 Pesaro, Italy; [2]Medical Oncology Unit, Ospedale di Fabriano, 60044 Fabriano, Italy; [3]Department of Radiation Oncology, Ospedale S Gerardo, 20052 Monza, Italy; [4]Division of Medical Oncology, Ospedale S Carlo Borromeo, 20153 Milano, Italy; [5]Medical Oncology Unit, Ospedale S Paolo, 20153 Milano, Italy; [6]Division of Medical Oncology, Ospedale L Sacco, 20157 Milano, Italy; [7]Medical Oncology, Casa di Cura Igea, 20153 Milano, Italy; [8]Division of Medical Oncology, Ospedale Civile, 15100 Alessandria, Italy; [9]Division of Medical Oncology, Ancona University, 60020 Ancona, Italy

**Summary** In a randomized clinical trial, gemcitabine (GEM) was more effective than 5-fluorouracil (5-FU) in advanced pancreatic cancer patients. GEM and 5-FU have different mechanisms of action and their combination, from a theoretical point of view, could result in a higher activity. To test activity and feasibility of such a combination, a multi-institutional phase II study was initiated in November 1996 by the Italian Group for the study of Digestive Tract Cancer (GISCAD). Primary objectives of this study were to determine the activity in terms of response rate and clinical benefit, while the secondary objective was toxicity. According to the optimal two-stage phase II design, 54 patients were enrolled. Schedule was: GEM 1000 mg $m^{-2}$ intravenous (i.v.), and 5-FU 600 mg $m^{-2}$ bolus i.v. weekly for 3 weeks out of every 4. All the 54 patients were symptomatic (pain, weight loss, dyspepsia). A clinical benefit was obtained in 28 patients (51%) (95% confidence interval (CI) 38–64%). Two patients achieved a partial response and 34 a stable disease. Median survival for all the patients was 7 months. Side-effects were mild: no gastrointestinal or haematological grade 3–4 toxicity (WHO) were recorded. We observed only six episodes of grade 2 (WHO) leukopenia and seven episodes of thrombocytopenia. Although the non-randomized design of this study suggests caution in the interpretation of these data, in consideration of the low incidence of toxicity and the favourable results obtained in terms of clinical benefit, it may be worthwhile to test more active schedules of 5-FU (continuous infusion) in combination with gemcitabine.

**Keywords**: pancreatic cancer; intensive chemotherapy; palliation

Pancreatic cancer is a rapidly fatal disease, with a 5-year survival rate of less than 5% (Parker et al, 1997). Surgery has been considered the only curative modality for this disease even if at the time of diagnosis the majority of patients have locally advanced unresectable or metastatic disease (Kelly, 1995).

Until very recently, chemotherapy was held to be largely ineffective in terms of objective responses, survival or quality of life in advanced pancreatic cancer patients (Taylor, 1993; Lionetto et al, 1995). However, opinions about the value of chemotherapy in advanced pancreatic cancer are changing. Cautious optimism for systemic chemotherapy is growing. This has been prompted by new drugs and new treatment end points as quality of life and symptom palliation. In fact, recent clinical trials with new drugs that have included analysis of clinical benefit end points have confirmed that chemotherapy is worthwhile and it can represent an important tool for improving the patient conditions (Ahlgren, 1996; Schnall et al, 1996; Popescu et al, 1997).

Gemcitabine (GEM), a novel nucleoside analogue, is the most investigated new drug in pancreatic cancer (Grindey et al, 1990). In a randomized clinical trial, GEM was more effective than 5-fluorouracil (5-FU) (given at a dose of 600 mg $m^{-2}$ intravenously (i.v.) over 30 min, with the cycle defined as one 4-week period) in alleviation of some disease-related symptoms and in survival benefit for patients with advanced pancreatic cancer (Burris et al, 1997).

Because GEM and 5-FU work in different ways to inhibit DNA and RNA synthesis and, furthermore, one of the effects of GEM, the inhibition of ribonucleotide reductase, could lead to an increased 5-FU activity (Heinemann et al, 1991), a study was performed to determine if the combination of both drugs can improve upon the results obtained with GEM alone.

*Received 22 July 1998*
*Revised 10 December 1998*
*Accepted 2 April 1999*

*Correspondence to:* S Cascinu

### PATIENTS AND METHODS

### Patient selection

Patients with histologically verified, locally advanced unresectable and/or metastatic pancreatic carcinoma were eligible for the study. Other eligibility criteria included: measurable disease, Karnofsky performance status (KPS) 60–90, age less than 70 years, normal liver (serum bilirubin < 1.5 mg $dl^{-1}$), renal (serum creatinine < 1.5 mg $dl^{-1}$) and bone marrow (leucocyte count > 4000 $\mu l^{-1}$, platelet count > 100 000 µl) functions.

Patients were excluded if they had undergone previous chemotherapy. Patients who had had radiotherapy to individual sites of disease were eligible but the disease site(s) was considered non-evaluable for response.

Informed consent was obtained from all participants after the nature of the study had been fully explained and the protocol was approved by the institutional review board.

### Chemotherapy

GEM was diluted in normal saline and administered i.v. over 30 min at the dose of 1000 mg $m^{-2}$. 5-FU was given as an i.v. bolus at the dose of 600 mg $m^{-2}$. Both drugs were administered once weekly for 3 consecutive weeks out of every 4 weeks.

Treatment with GEM and 5-FU continued until there was evidence of disease progression, or until there was significant clinical deterioration because of tumour-related symptoms. Patients were not allowed to receive concomitant radiation therapy, chemotherapy, hormonal therapy or corticosteroids during the trial.

### Efficacy and safety evaluation

### *Clinical benefit*

The principal efficacy end point used in this study was clinical benefit derived from measurement of three common debilitating signs or symptoms present in most patients with advanced pancreas cancer: pain, functional impairment and weight loss (Moore, 1996; Rothenberg et al, 1996*a*).

Pain assessment was comprised of separate measures of both pain intensity and analgesic consumption. Each patient was categorized as positive, negative or stable for these two pain-related outcomes. Pain intensity score as the 100 mm visual analogue scale (0 = least possible pain to 100 = worst possible pain) was recorded in a patient card completed daily. The weekly pain intensity score was computed as the mean of the daily pain intensity scores of the preceding 7 days. A positive change in pain intensity was defined as at least 4 consecutive weeks during which the pain intensity measurements were ≥ 50% improved from baseline. A negative change was defined as either ≥ 4 consecutive weeks with pain intensity measurements that were worse than baseline by any degree, or discontinuation from study within the first 12 weeks of treatment due to increasing pain. Pain intensity was considered stable if the criteria for positive or negative change were not met. Analgesic consumption was computed on a weekly basis as the mean of the daily analgesic consumption (expressed in terms of morphine equivalent mg per day) for the preceding 7 days. A positive change in analgesic consumption was defined as ≥ 4 consecutive weeks with analgesic consumption ≤ 50% compared to baseline. Negative change in analgesic consumption was defined as ≥ 4 consecutive weeks with analgesic consumption that was worse than baseline by any degree.

KPS was determined by two independent observers at baseline and on a weekly basis thereafter. In cases where the scores differed, the lower of the two scores was scheduled.

Clinical benefit response was determined by both pain assessment classification and KPS. A patient was considered to be a clinical benefit non-responder by the primary measures of response if either pain or KPS was classified as negative. Barring this occurrence, if either the pain or performance status measures were positive a patient was identified as a clinical benefit responder. If pain and performance status were both stable, then the secondary measure of weight change was used to determine clinical benefit. A patient's weight was recorded once at baseline and weekly thereafter. If the patient developed third-space fluid or required parenteral nutrition at any time during the study, the patient was considered non-positive for weight change. Positive weight change was defined as an increase in weight by ≥ 7% over baseline that was sustained for at least 4 consecutive weeks. Any other occurrence was defined as a non-positive change in weight. Patients who were stable on pain assessment and performance status were considered to be clinical benefit responders if they experienced a positive change in weight.

Patients were categorized as clinical benefit non-responders if they were stable on the primary measures and experienced a non-positive change in weight.

For a patient to be classified as clinical benefit responder, at least one component of clinical benefit response (e.g. pain, KPS or weight change) must have been positive with none of the other components negative.

### *Other measures of efficacy*

In addition to the clinical benefit measurement, objective tumour response and survival were assessed prospectively as additional end points. Disease status for patients was assessed every 8 weeks.

### *Safety*

Patients were evaluated by weekly history and physical examinations, including complete blood counts and chemistry profile. All signs, symptoms or laboratory abnormalities were assessed using WHO criteria for toxicities (Miller et al, 1981).

### Statistical methods

This was a multi-institutional phase II study; the primary objective was to determine the activity of this combination (clinical benefit, response rate) while secondary objective was to determine toxicity. According to the optimal two-stage phase II design the treatment programme was designed to reject a clinical benefit less than 20% (p0) and to provide a statistical power of 90% in assessing the activity of the regimen (in terms of response rate) as 40% (p1) (p1–p0 = 20%) for an $\alpha$ error less than 0.05 (Simon, 1989).

The 95% exact confidence interval (CI) for response was calculated. Survival time was calculated from the onset of chemotherapy.

### RESULTS

Investigators from nine GISCAD (Italian Group for the Study of Digestive Tract Cancer) centres treated 54 advanced pancreatic cancer patients with this weekly regimen between November 1996 and August 1997. The median follow-up from the start of treatment was 10 months (range 6–14 months). The characteristics of treated patients are detailed in Table 1. Thirty-eight patients demonstrated a progressive disease in the 2 months before the onset of chemotherapeutic treatment, either clinically or radiologically. None of the patients received radiation therapy for primary tumour or metastatic disease.

### Clinical benefit

Twenty-eight patients were classified as positive in the pain category (i.e. pain intensity and/or analgesic use was reduced). In ten patients both pain and KPS improved, while in the other 18 there was an improvement in pain with no worsening of

*© 1999 Cancer Research Campaign*

**Table 1** Patient characteristics

| | |
|---|---|
| No. of patients | 54 |
| Age (years) | |
| Median | 56 |
| Range | 47–70 |
| Sex | |
| M/F | 36/18 |
| Performance status (Karnofsky index) | |
| 80–90 | 14 |
| 60–70 | 40 |
| Disease at presentation | |
| Locally advanced | 25 |
| Metastatic | 10 |
| Locally advanced and metastatic | 19 |
| Site of primary tumour | |
| Head | 38 |
| Body | 12 |
| Tail | 4 |
| Histology | |
| Well-differentiated | 15 |
| Moderately differentiated | 28 |
| Poorly differentiated | 11 |
| Baseline pain intensity score | |
| Median | 45 |
| Range | 25–80 |
| Median baseline analgesic requirement (morphine equivalent mg day$^{-1}$) | 90 |
| Previous treatments | |
| Surgery | 15 |
| Radiotherapy | 0 |

performance status. Therefore, these patients were classified as clinical benefit responders by their primary measures.

With regard to the secondary measure of clinical benefit, seven patients had a positive weight change. All of them had already been categorized as clinical benefit responders by primary measures. The other 21 patients achieving a clinical benefit, i.e. had a stable weight.

The median time to achieve a clinical benefit response was 4 weeks, while the median duration of clinical benefit was 19 weeks.

### Tumour response

All patients had bidimensionally measurable disease on computerized tomography (CT) scan at study entry. Two patients achieved a partial response, ten patients a minor response, while 24 had a stable disease. The time to progression was 3.4 months. The median survival was 7 months. Patients with partial response and stable disease had a median survival of 9 months. The estimated survival rate beyond 6 months was 61%, beyond 9 months was 35% and beyond 12 months was 22%.

### Toxicity

Side-effects were mild: no gastrointestinal or haematological grade 3–4 WHO toxicities were observed. We recorded only six episodes of grade 2 leukopenia and seven episodes of grade 2 thrombocytopenia. We did not observe any treatment-related death. Specific treatment-related toxicities are detailed in Table 2. There was no cumulative toxicity in the following treatment weeks.

**Table 2** Maximum toxicity observed for each patient

| | WHO grade | | | |
|---|---|---|---|---|
| | 1 | 2 | 3 | 4 |
| Leukopenia | 4 | 6 | – | – |
| Thrombocytopenia | 7 | 7 | – | – |
| Anaemia | 6 | 2 | – | – |
| Diarrhoea | 2 | – | – | – |
| Nausea/vomiting | 4 | 2 | – | – |

### DISCUSSION

In patients with pancreatic carcinoma, several chemotherapeutic regimens have been tested with limited impact on patient outcome. However, the development of new drugs and the introduction of innovative end points of treatment have led to a cautious optimism for the use of chemotherapy for advanced disease. In particular, GEM, camptothecin derivatives such as topotecan or irinotecan, as well as taxanes have been investigated for toxicity and activity in advanced pancreatic cancer (Casper et al, 1994; Rougier et al, 1994; Carmichael et al, 1995; Wagener et al, 1995; Rothenberg et al, 1996*b*; Scher et al, 1996; Whitehead et al, 1997).

GEM showed a very favourable toxicity profile and demonstrated activity in advanced pancreatic cancer. It is a nucleoside analogue, inhibiting DNA synthesis by the accumulation of difluorodeoxycitidine triphosphate, which competes with deoxycitidine triphosphate for incorporation into DNA (Grindey et al, 1990). Furthermore, GEM seems to be a potent inhibitor of ribonucleotide reductase. The inhibition of ribonucleotide reductase limits the production of deoxyuridine monophosphate (Heinemann et al, 1991). Because this effect can enhance 5-FU activity, by decreasing the competition with fluorodeoxyuridine monophosphate, a combination of 5-FU with GEM can improve cell cytotoxicity.

We decided to combine GEM and 5-FU at the dose used in the randomized trial by Burris et al (1997). The dose of 5-FU (600 mg m$^{-2}$) was not the maximum possible for treatment of patients with pancreas cancer, but there is no evidence that either a higher dose of 5-FU or modulation with leucovorin are more effective (DeCaprio et al, 1991; Schnall et al, 1996). The schedule of GEM was, in part, minimally modified with respect to that proposed by Burris et al (1997), starting with the administrations once a week for 3 consecutive weeks out of every 4 weeks. In fact, in our previous experience on five patients treated with a weekly administration for 7 consecutive weeks, the fourth administration was always delayed (data not shown).

Tumour response rate was low (3.7%) and similar to that obtained with GEM alone in the randomized trial. A minor response was observed in ten patients. In spite of this low objective response rate, 51% of patients treated in our study responded to chemotherapy by achieving sustained improvement in pain, KPS, or both. A clinical benefit was achieved in all the patients with a partial or minor response, as well as in 16 patients with stable disease, suggesting that in patients with advanced pancreatic cancer a less than 50% decrease in measurable tumour bulk, or even objectively measured stable disease, after treatment may be associated with a clinical benefit. The discrepancy between objective tumour response and clinical benefit could be due to the difficulty to quantify the true proportion of tumour response in

*© 1999 Cancer Research Campaign*

pancreatic tumour. In fact, since the important desmoplastic reaction induced by the tumour, including inflammation and fibrosis within and around the tumour, does not necessarily shrink after chemotherapy, the size of tumour on a CT scan may not reflect the true proportion of tumour response (Ahlgren, 1996). Therefore, the use of clinical benefit as primary end point may be more useful than tumour objective response in assessing the activity of chemotherapy in pancreatic cancer.

This higher incidence of clinical benefit respect to that reported in Burris's study seems to indicate that the combination of GEM and 5-FU could be more effective than GEM alone. Furthermore, the survival rates at 6, 9 and 12 months were better in our study than those reported in Burris's study: 61%, 35% and 22% versus 46%, 24% and 18% respectively.

Therapy was well tolerated with no evidence of grade 3–4 toxicities. It could be due to the modification of the schedule. In our previous experience using the schedule proposed by Burris et al (1997), most of the side-effects occur at week 5–6 of the first part of treatment (7 consecutive weeks) and much less in the second part of the therapeutic programme (once weekly for 3 out of every 4 weeks).

In conclusion, although the non-randomized design suggests caution in the interpretation of results, considering the low incidence of toxicity and the better results obtained in terms of clinical benefit, it may be worthwhile to test more active schedules of 5-FU (continuous infusion) in combination with GEM.

### REFERENCES

Ahlgren JD (1996) Chemotherapy for pancreatic cancer. *Cancer* **78**: 654–663

Burris III HA, Moore MJ, Andersen J, Green MR, Rothenberg ML, Modiano MR, Cripps MC, Portenoy RK, Storniolo AM, Tarassoff P, Nelson R, Dorr FA, Stephens CD and Von Hoff DD (1997) Improvements in survival and clinical benefit with gemcitabine as first-line therapy for patients with advanced pancreas cancer: a randomized trial. *J Clin Oncol* **15**: 2403–2413

Carmichael J, Fink U, Russel RCG, Spittle MF, Harris A and Spiessl G (1995) Phase II study of gemcitabine in patients with advanced pancreatic cancer. *Br J Cancer* **73**: 101–105

Casper ES, Green MR, Kelsen DP, Heelan RT, Brown TD and Flombaum CD (1994) Phase II trial of gemcitabine in patients with adenocarcinoma of the pancreas. *Invest New Drug* **12**: 29–35

DeCaprio JA, Mayer RJ, Gonin R and Arbuck SG (1991) Fluorouracil and high-dose leucovorin in previously untreated patients with advanced adenocarcinoma of the pancreas: results of a phase II trial. *J Clin Oncol* **9**: 2128–2133

Grindey GB, Hertel LW and Plunkett W (1990) Cytotoxicity and antitumor activity of 2′,2′-difluorodeoxycitidine (Gemcitabine). *Cancer Invest* **8**: 313–318

Heinemann V, Xu YZ, Chubb S, Sen A, Hertel LW, Grindey GB and Plunkett W (1991) Inhibition of ribonucleotide reductase in CCRF-CEM cells by 2′,2′-difluorodeoxycitidine. *Mol Pharmacol* **38**: 567–572

Kelly DM and Benjamin IS (1995) Pancreatic carcinoma. *Ann Oncol* **6**: 19–28

Lionetto R, Pugliese V, Bruzzi P and Rosso R (1995) No standard treatment is available for advanced pancreatic cancer. *Eur J Cancer* **6**: 882–887

Miller AB, Hoodgstraten B, Staquet M and Winkler A (1981) Reporting results of cancer treatment. *Cancer* **47**: 207–214

Moore M (1996) Activity of gemcitabine in patients with advanced pancreatic carcinoma. A review. *Cancer* **78**: 633–638

Parker SL, Tong T, Bold S and Wingo PA (1997) Cancer statistics, 1997. *CA Cancer Clin* **47**: 5–27

Popescu RA and Cunningham D (1997) Chemotherapy for advanced pancreatic cancer. Some lights at the end of the tunnel? *Ann Oncol* **8**: 415–416

Rothenberg ML, Abbruzzese JL, Moore M, Portenoy RK, Robertson JM and Wanebo HJ (1996) A rationale for expanding the endpoints for clinical trials in advanced pancreatic carcinoma. *Cancer* **78**: 627–632

Rothenberg LM, Moore MJ, Cripps MC, Andersen JS, Portenoy RK, Burris III HA, Green MR, Tarassoff PG, Brown TD, Casper ES, Storniolo AM and Von Hoff DD (1996) A phase II trial of gemcitabine in patients with 5FU-refractory pancreas cancer. *Ann Oncol* **7**: 347–353

Rougier P, De Forni M, Adenis A, Ducreux M, Djazouli K, Adams D, Bonneterre J, Clouet P, Blanc C, Bayssas M and Armand JP (1994) Phase II study of taxotere in pancreatic adenocarcinoma. *Proc Am Soc Clin Oncol* **13**: 200

Schnall S and Macdonald JS (1996) Chemotherapy of adenocarcinoma of the pancreas. *Semin Oncol* **23**: 220–228

Scher MN, Kosierowski R, Lusch C, Alexander R, Fox S, Redei I, Green F, Raskay B, Amfoh K, Engstrom PF and O'Dwyer PJ (1996) Phase II trial of topotecan in advanced or metastatic adenocarcinoma of the pancreas. *Invest New Drugs* **13**: 347–354

Simon R (1989) Optimal two-stage designs for phase II clinical trials. *Control Clin Trials* **10**: 1–10

Taylor I (1993) Should further studies of chemotherapy be carried out in pancreatic cancer? *Eur J Cancer* **29**: 1076–1078

Wagener DJT, Verdonk HER, Dirix LY, Catimel G, Siegenthaler P, Buitenhuis M, Mathieu-Boué A and Verweij J (1995) Phase II trial of CPT11 in patients with advanced pancreatic cancer, an EORTC early clinical trial group study. *Ann Oncol* **6**: 129–132

Whitehead RP, Jacobson J, Brown TD, Taylor SA, Weiss GR and Macdonald JS (1997) Phase II trial of paclitaxel and granulocyte colony-stimulating factor in patients with pancreatic carcinoma: A Southwest Oncology Group Study. *J Clin Oncol* **15**: 2414–2419

*© 1999 Cancer Research Campaign*
